# Supplementary material for: LncRNA LINC01537 Promotes Gastric Cancer Metastasis and Tumorigenesis by Stabilizing RIPK4 to Activate NF-κB Signaling
Source: Cancers (Basel). 2022 Oct 25;14(21):5237. doi: 10.3390/cancers14215237 (PMC9657364; doi:10.3390/cancers14215237)
Supplement: Supplementary file 1 [file cancers-14-05237-s001.zip › Figure S legend.pdf]

Figure S1. Localization, length and encoded protein potential of LINC01537.
A. LINC01537 is located at chr11:72,281,700-72,284,266 (https://genome-asia.ucsc.edu/). B. LINC01537 consists of 2567bp(shown on NCBI, https://www.ncbi.nlm.nih.gov/). C. Online Coding Potential Assessment Tool(CPAT, https://lilab.research.bcm.edu/cpat/index.php) confirmed that LINC01537 did not have the ability to encode a protein.

Figure S2. qRT-PCR results revealed that the knockdown efficiency of these two siRNAs was approximately 70%. One-way ANOVA was employed to analyze the statistical differences. **** $P < 0.0001$.

Figure S3. LINC01537 knockdown did not inhibits proliferation, migration, and invasion of GES-1. After knockdown and overexpression of LINC01537 in normal gastric epithelial cell line GES-1, CCK8 assays and Transwell assays were conducted. We found that after knockdown of LINC01537, there was no significant change in the proliferation, invasion and migration ability of GES-1. However, the overexpression of LINC01537 significantly enhanced the proliferation, invasion and migration ability of GES-1

Figure S4. The levels of β-catenin, RAF1/MEK/ERK and STAT3 did not change significantly when knockdown LINC01537.

Figure S5. qRT-PCR results revealed that the mRNA levels of RIPK4 remained unchanged when LINC01537 was knocked down. One-way ANOVA was employed to analyze the statistical differences.

Figure S6. qRT-PCR and western blot results revealed that the mRNA and protein levels of Trim25 remained unchanged when LINC01537 was knocked down. One-way ANOVA was employed to analyze the statistical differences.

Figure S7. Western blot results revealed that the protein levels of RIPK4 in si-NC or si-1 BGC-823 and AGS transfected with ectopic RIPK4-overexpression plasmid, or empty control.

Figure S8. The protein level of NF-κB decreased after knockdown of LINC01537. After replenishing RIPK4, the decreased ptotein level of NF-κB was completely relieved.
