# Supplementary material for: LncRNA LINC01537 Promotes Gastric Cancer Metastasis and Tumorigenesis by Stabilizing RIPK4 to Activate NF-κB Signaling
Source: Cancers (Basel). 2022 Oct 25;14(21):5237. doi: 10.3390/cancers14215237 (PMC9657364; doi:10.3390/cancers14215237)
Supplement: Supplementary file 1 [file cancers-14-05237-s001.zip › Table S1.pdf]

Table S1: PCR Primer

	Sequence of Primer
GADPH-F	GGACCTGACCTGCCGTCTAG
GADPH-R	GTAGCCCAGGATGCCCTTGA
MALAT-1-F	GAATTGCGTCATTTAAAGCCTAGTT
MALAT-1-R	GTTTCATCCTACCACTCCCAATTAAT
LINC01537-F	CACTTGTGGCATTATGTCGGT
LINC01537-R	CCTCTTAGGTAGGAATCTGGGTA
RIPK4-F	TGAGTGTGAACGCCCAGA
RIPK4-R	GAGCACAGGTCGATGAGGA
NF-κB-p65-F	ATGTGGAGATCATTGAGCAGC
NF-κB-p65-R	CCTGGTCCTGTGTAGCCATT
Trim25-F	AGCAGCTACAACAAGAATACACG
Trim25-R	GGCTCTGTTCAATCTCCTCCT

F:Forward primer; R:Reverse primer
