# Supplementary material for: LncRNA LINC01537 Promotes Gastric Cancer Metastasis and Tumorigenesis by Stabilizing RIPK4 to Activate NF-κB Signaling
Source: Cancers (Basel). 2022 Oct 25;14(21):5237. doi: 10.3390/cancers14215237 (PMC9657364; doi:10.3390/cancers14215237)
Supplement: Supplementary file 1 [file cancers-14-05237-s001.zip › Table S10.pdf]

Table S10: Source data for Figure 4M

Target	NC	Si-1	Si-2
IP Trim25	24619	46721	38517
IP IgG	1371	1061	1052
Input	48172	52719	43171
IP Trim25	47267	50228	47876

Data shown as Integrated Gray Density.
