# Supplementary material for: LncRNA LINC01537 Promotes Gastric Cancer Metastasis and Tumorigenesis by Stabilizing RIPK4 to Activate NF-κB Signaling
Source: Cancers (Basel). 2022 Oct 25;14(21):5237. doi: 10.3390/cancers14215237 (PMC9657364; doi:10.3390/cancers14215237)
Supplement: Supplementary file 1 [file cancers-14-05237-s001.zip › Table S11.pdf]

Table S11: Source data for Figure 6A

Target	NC	NC+inhibitor	OE	OE+inhibitor
NF-kB-p65	19358/0.51	13250/0.36	37096/1.08	17226/0.51
GADPH	38027	36721	34271	33916

Data shown as Integrated Gray Density or Integrated Gray Density/ relative expression.
