# Supplementary material for: LncRNA LINC01537 Promotes Gastric Cancer Metastasis and Tumorigenesis by Stabilizing RIPK4 to Activate NF-κB Signaling
Source: Cancers (Basel). 2022 Oct 25;14(21):5237. doi: 10.3390/cancers14215237 (PMC9657364; doi:10.3390/cancers14215237)
Supplement: Supplementary file 1 [file cancers-14-05237-s001.zip › Table S12.pdf]

Table S12: Source data for Figure S4

Target	Left panel			Right panel		
	NC	Si-1	Si-2	NC	Si-1	Si-2
RAF-1	30172/0.61	35821/0.81	39922/0.88			
p-RAF-1	39271/0.80	31817/0.72	42725/0.94			
MEK	42179/0.86	45217/1.02	43216/0.96			
p-MEK	49281/1.00	42716/0.97	47852/1.06			
ERK	38261/0.78	34726/0.78	39271/0.87			
STAT3				48171/0.92	43169/0.88	44662/0.97
b-catenin				38628/0.74	42818/0.87	39715/0.86
GADPH	49172	44261	45214	52174	49217	46215

Data shown as Integrated Gray Density or Integrated Gray Density/Relative expression.
