# Supplementary material for: LncRNA LINC01537 Promotes Gastric Cancer Metastasis and Tumorigenesis by Stabilizing RIPK4 to Activate NF-κB Signaling
Source: Cancers (Basel). 2022 Oct 25;14(21):5237. doi: 10.3390/cancers14215237 (PMC9657364; doi:10.3390/cancers14215237)
Supplement: Supplementary file 1 [file cancers-14-05237-s001.zip › Table S13.pdf]

Table S13: Source data for Figure S6

Target	AGS			BGC-823		
	NC	Si-1	Si-2	NC	Si-1	Si-2
Trim25	57215/0.77	55681/0.77	49281/0.65	54271/0.87	56214/0.80	53095/0.85
GADPH	74261	72514	75526	62319	70168	62184

Data shown as Integrated Gray Density or Integrated Gray Density/Relative expression.
