# Supplementary material for: LncRNA LINC01537 Promotes Gastric Cancer Metastasis and Tumorigenesis by Stabilizing RIPK4 to Activate NF-κB Signaling
Source: Cancers (Basel). 2022 Oct 25;14(21):5237. doi: 10.3390/cancers14215237 (PMC9657364; doi:10.3390/cancers14215237)
Supplement: Supplementary file 1 [file cancers-14-05237-s001.zip › Table S14.pdf]

Table S14: Source data for Figure S7

Target	AGS				BGC-823			
	NC+Vector	NC+RIPK4	Si-1+Vector	Si-1+RIPK4	NC+Vector	NC+RIPK4	Si-1+Vector	Si-1+RIPK4
RIPK4	25601/0.26	39525/0.43	18261/0.21	26505/0.32	30281/0.34	43625/0.44	22714/0.28	34271/0.40
GADPH	99826	92716	85171	82018	88271	98911	80144	86251

Data shown as Integrated Gray Density or Integrated Gray Density/Relative expression.
