# Supplementary material for: LncRNA LINC01537 Promotes Gastric Cancer Metastasis and Tumorigenesis by Stabilizing RIPK4 to Activate NF-κB Signaling
Source: Cancers (Basel). 2022 Oct 25;14(21):5237. doi: 10.3390/cancers14215237 (PMC9657364; doi:10.3390/cancers14215237)
Supplement: Supplementary file 1 [file cancers-14-05237-s001.zip › Table S15.pdf]

Table S15: Source data for Figure S8

Target	BGC-823			
	NC+Vector	NC+RIPK4	Si-1+Vector	Si-1+RIPK4
NF-kB-p65	17625/0.47	29149/0.69	7582/0.18	25686/0.66
GADPH	37261	42271	42591	39175

Data shown as Integrated Gray Density or Integrated Gray Density/Relative expression.
