# Supplementary material for: LncRNA LINC01537 Promotes Gastric Cancer Metastasis and Tumorigenesis by Stabilizing RIPK4 to Activate NF-κB Signaling
Source: Cancers (Basel). 2022 Oct 25;14(21):5237. doi: 10.3390/cancers14215237 (PMC9657364; doi:10.3390/cancers14215237)
Supplement: Supplementary file 1 [file cancers-14-05237-s001.zip › Table S2.pdf]

Table S2: SiRNA sequence

	sense（5’-3’）	antisense（5’-3’）
si-1	GCAUCUACUCACCAUCCUUTT	AAGGAUGGUGAGUAGAUGCTT
si-2	GCUGGAGGCCUCCAAGAAATT	UUUCUUGGAGGCCUCCAGCTT
