# Supplementary material for: LncRNA LINC01537 Promotes Gastric Cancer Metastasis and Tumorigenesis by Stabilizing RIPK4 to Activate NF-κB Signaling
Source: Cancers (Basel). 2022 Oct 25;14(21):5237. doi: 10.3390/cancers14215237 (PMC9657364; doi:10.3390/cancers14215237)
Supplement: Supplementary file 1 [file cancers-14-05237-s001.zip › Table S3.pdf]

Table S3: Source data for Figure 4C

Target	5% Input	Antisense LINC01537	LINC01537
RIPK4	45417/1.26	2148/3.58	38553/62.38
GADPH	36010	600	618

Data shown as Integrated Gray Density or Integrated Gray Density/ relative expression.
