# Supplementary material for: LncRNA LINC01537 Promotes Gastric Cancer Metastasis and Tumorigenesis by Stabilizing RIPK4 to Activate NF-κB Signaling
Source: Cancers (Basel). 2022 Oct 25;14(21):5237. doi: 10.3390/cancers14215237 (PMC9657364; doi:10.3390/cancers14215237)
Supplement: Supplementary file 1 [file cancers-14-05237-s001.zip › Table S4.pdf]

**Table S4: Source data for Figure 4D**

| Target | F1 | F2 | F3 | F4 | F5 | F6 |
|---|---|---|---|---|---|---|
| RIPK4 | 28296 | 9081 | 11746 | 34192 | 4934 | 5683 |

Data shown as Integrated Gray Density.
