# Supplementary material for: LncRNA LINC01537 Promotes Gastric Cancer Metastasis and Tumorigenesis by Stabilizing RIPK4 to Activate NF-κB Signaling
Source: Cancers (Basel). 2022 Oct 25;14(21):5237. doi: 10.3390/cancers14215237 (PMC9657364; doi:10.3390/cancers14215237)
Supplement: Supplementary file 1 [file cancers-14-05237-s001.zip › Table S5.pdf]

Table S5: Source data for Figure 4F

Target	AGS			BGC-823		
	NC	Si-1	Si-2	NC	Si-1	Si-2
NF-Kb-p65	49805/1.09	25258/0.54	14842/0.30	57553/1.14	24537/0.44	22579/0.41
RIPK4	48411/1.06	18090/0.39	22615/0.46	47661/0.94	32132/0.58	33190/0.61
GADPH	45527	46892	49297	50510	55195	54753

Data shown as Integrated Gray Density or Integrated Gray Density/Relative expression.
