# Supplementary material for: LncRNA LINC01537 Promotes Gastric Cancer Metastasis and Tumorigenesis by Stabilizing RIPK4 to Activate NF-κB Signaling
Source: Cancers (Basel). 2022 Oct 25;14(21):5237. doi: 10.3390/cancers14215237 (PMC9657364; doi:10.3390/cancers14215237)
Supplement: Supplementary file 1 [file cancers-14-05237-s001.zip › Table S6.pdf]

Table S6: Source data for Figure 4G

Target			AGS			
	MG132			DMSO		
	NC	Si-1	Si-2	NC	Si-1	Si-2
RIPK4	32070/0.95	29026/0.85	19265/0.54	28790/0.75	6460/0.15	10530/0.26
GADPH	33626	34102	35323	38229	41049	40544
			BGC-823			
	MG132			DMSO		
	NC	Si-1	Si-2	NC	Si-1	Si-2
RIPK4	27864/1.14	24398/0.73	24791/0.83	24177/0.90	14624/0.43	14443/0.41
GADPH	24505	33294	30081	26945	34392	35381

Data shown as Integrated Gray Density or Integrated Gray Density/Relative expression.
