# Supplementary material for: LncRNA LINC01537 Promotes Gastric Cancer Metastasis and Tumorigenesis by Stabilizing RIPK4 to Activate NF-κB Signaling
Source: Cancers (Basel). 2022 Oct 25;14(21):5237. doi: 10.3390/cancers14215237 (PMC9657364; doi:10.3390/cancers14215237)
Supplement: Supplementary file 1 [file cancers-14-05237-s001.zip › Table S7.pdf]

Table S7: Source data for Figure 4H

Target	AGS							
	NC				Si-1			
	0h	12h	24h	36h	0h	12h	24h	36h
RIPK4	36516/0.95	19704/0.57	13307/0.38	7217/0.18	30018/1.00	12076/0.33	10068/0.29	1972/0.05
GADPH	38454	34621	35429	39011	30106	36717	34131	37935
	BGC-823							
	NC				Si-1			
	0h	12h	24h	36h	0h	12h	24h	36h
RIPK4	34376/0.79	18914/0.48	13400/0.33	13516/0.33	37226/0.96	24604/0.65	11257/0.28	3512/0.09
GADPH	43537	39122	41131	41089	38908	37622	40442	39062

Data shown as Integrated Gray Density or Integrated Gray Density/Relative expression.
