# Supplementary material for: LncRNA LINC01537 Promotes Gastric Cancer Metastasis and Tumorigenesis by Stabilizing RIPK4 to Activate NF-κB Signaling
Source: Cancers (Basel). 2022 Oct 25;14(21):5237. doi: 10.3390/cancers14215237 (PMC9657364; doi:10.3390/cancers14215237)
Supplement: Supplementary file 1 [file cancers-14-05237-s001.zip › Table S8.pdf]

Table S8: Source data for Figure 4I

Target	AGS			BGC-823		
	NC	Si-1	Si-2	NC	Si-1	Si-2
Ub	132613/2.89	231461/4.41	223186/4.37	125861/2.43	226188/4.76	235161/4.99
GADPH	45848	52542	51046	51610	47518	47148

Data shown as Integrated Gray Density or Integrated Gray Density/Relative expression.
