# Supplementary material for: LncRNA LINC01537 Promotes Gastric Cancer Metastasis and Tumorigenesis by Stabilizing RIPK4 to Activate NF-κB Signaling
Source: Cancers (Basel). 2022 Oct 25;14(21):5237. doi: 10.3390/cancers14215237 (PMC9657364; doi:10.3390/cancers14215237)
Supplement: Supplementary file 1 [file cancers-14-05237-s001.zip › Table S9.pdf]

Table S9: Source data for Figure 4L

Target	Input	IgG	IP
RIPK4	34806	3033	38348
Trim25	58418	3724	42816

Data shown as Integrated Gray Density.
