# Supplementary figures and images for: LncRNA LINC01537 Promotes Gastric Cancer Metastasis and Tumorigenesis by Stabilizing RIPK4 to Activate NF-κB Signaling
Source: Cancers (Basel). 2022 Oct 25;14(21):5237. doi: 10.3390/cancers14215237 (PMC9657364; doi:10.3390/cancers14215237)

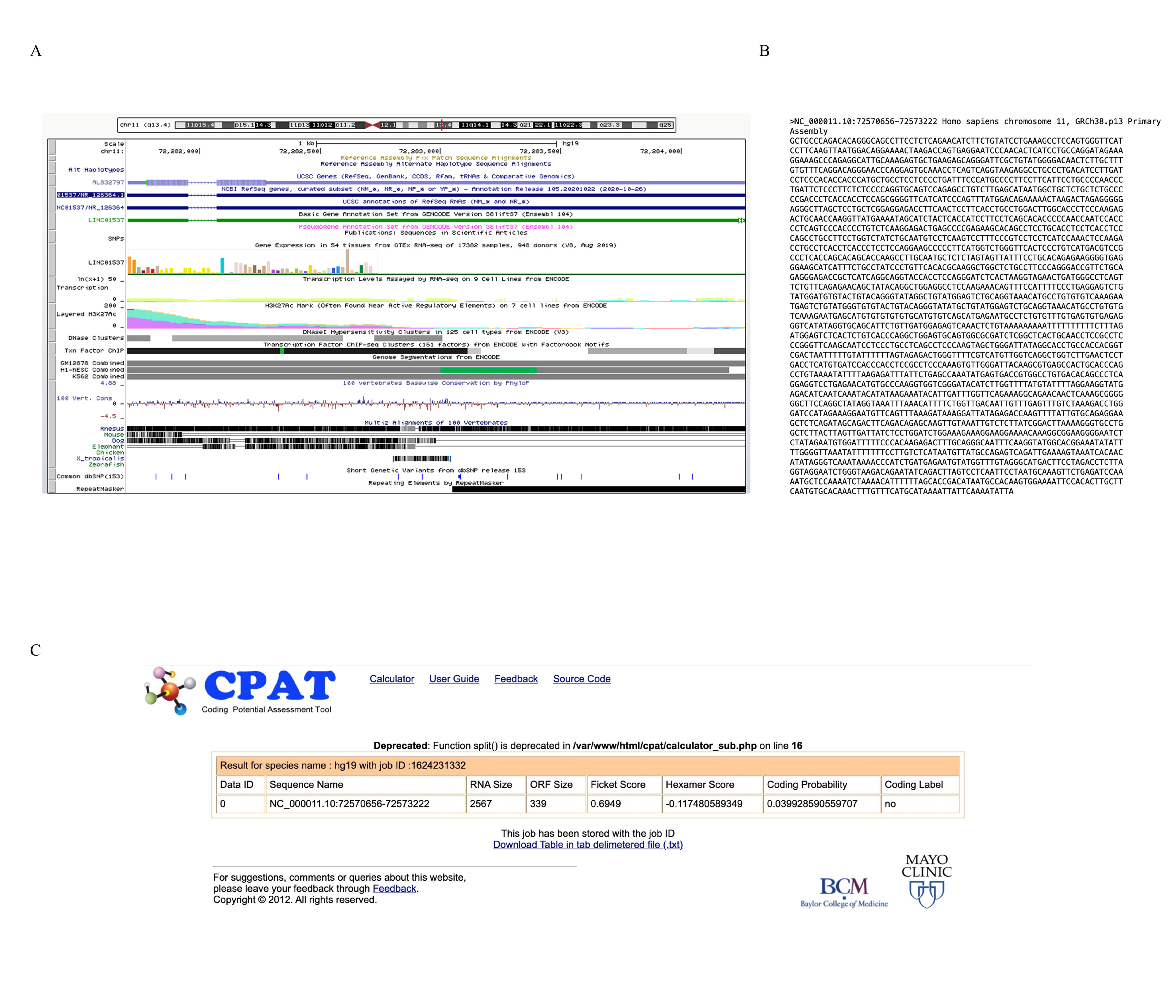

Supplement: Supplementary file 1 [file cancers-14-05237-s001.zip › Figure S1.tif]

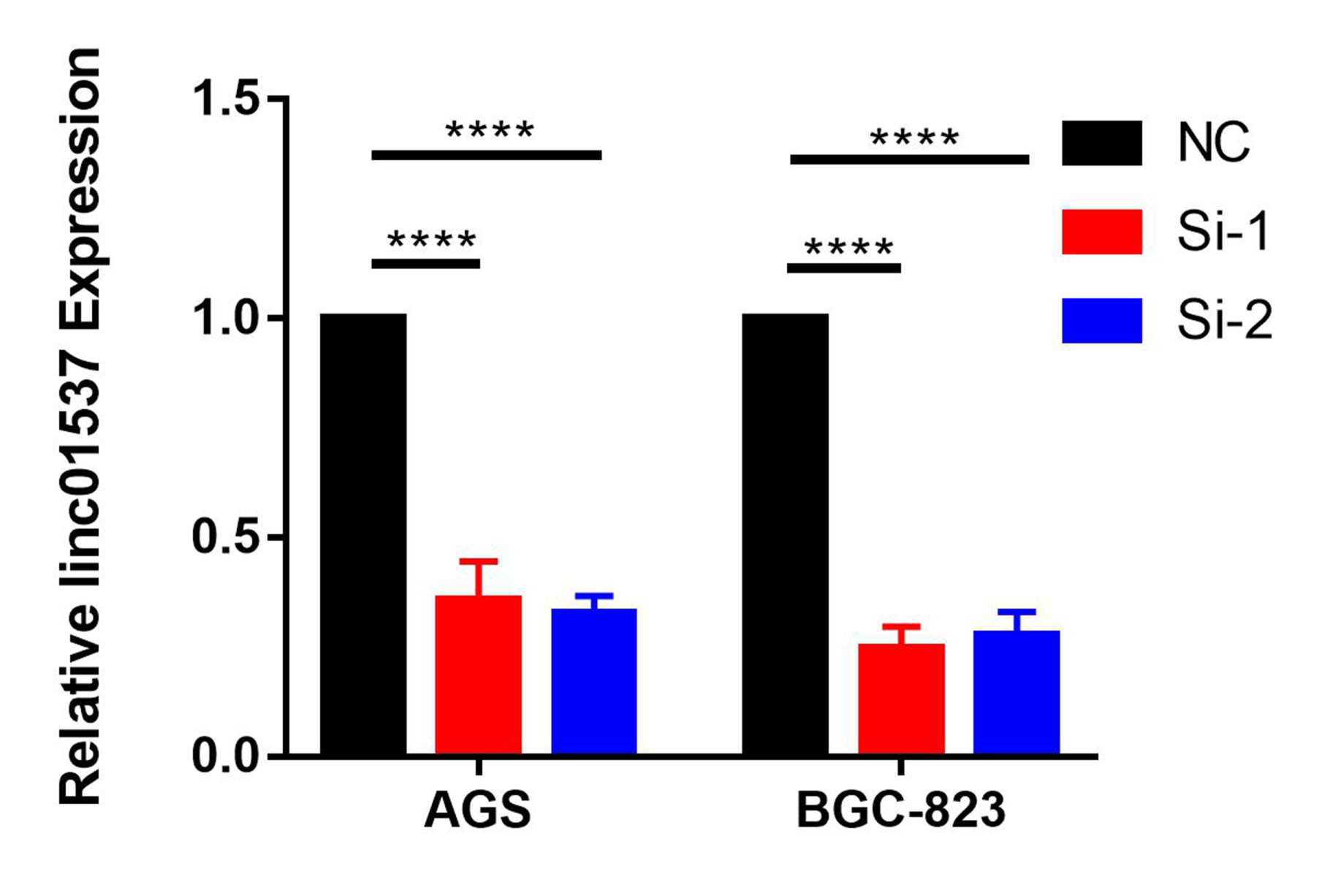

Supplement: Supplementary file 1 [file cancers-14-05237-s001.zip › Figure S2.tif]

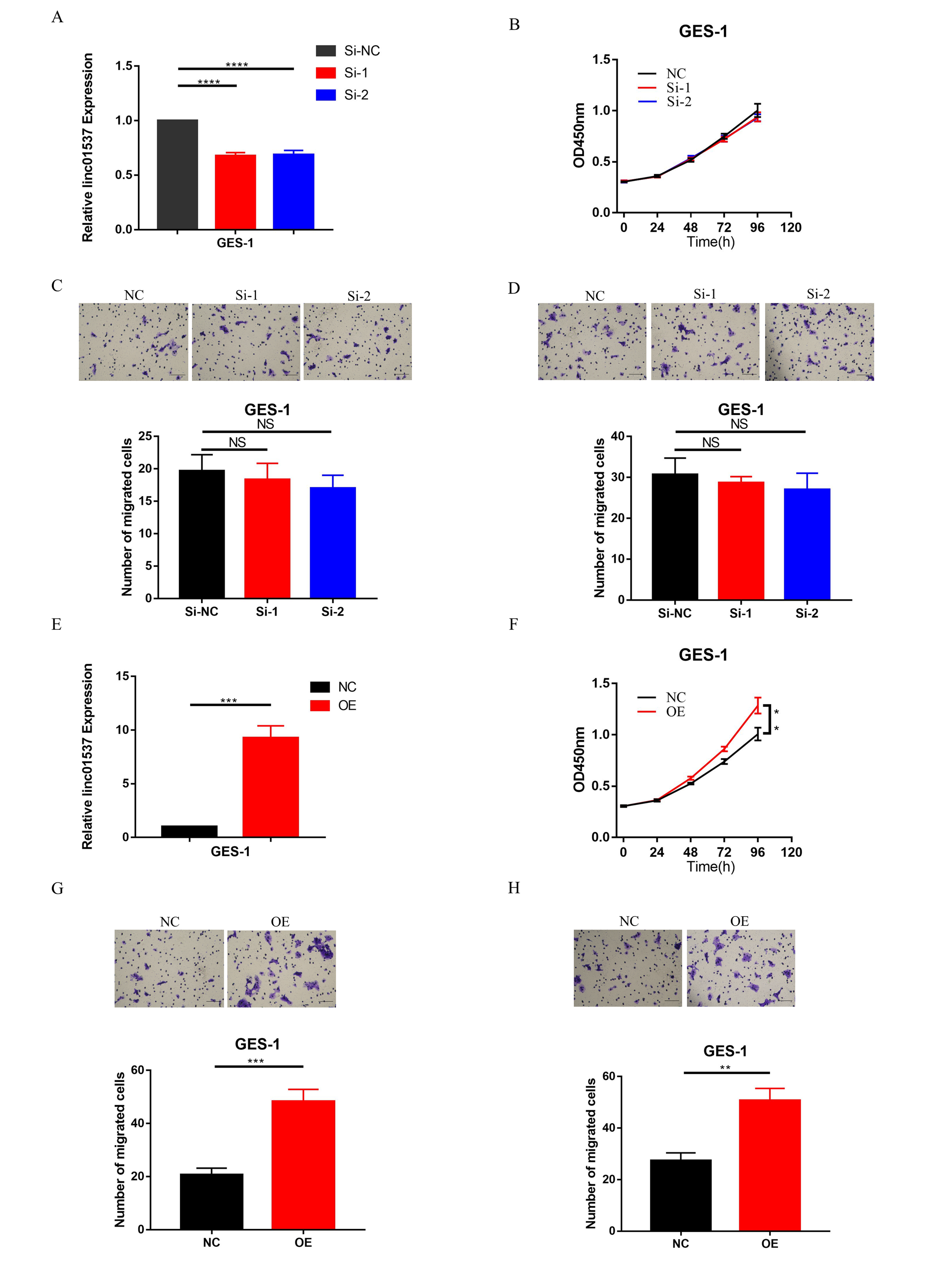

Supplement: Supplementary file 1 [file cancers-14-05237-s001.zip › Figure S3.tif]

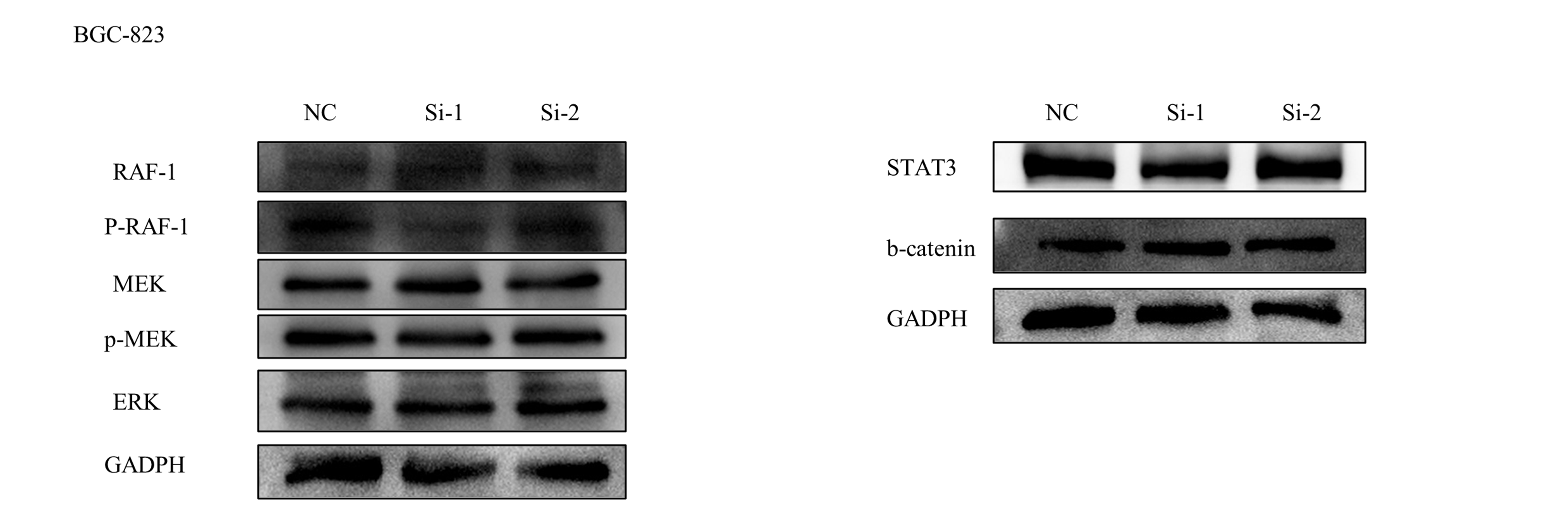

Supplement: Supplementary file 1 [file cancers-14-05237-s001.zip › Figure S4.tif]

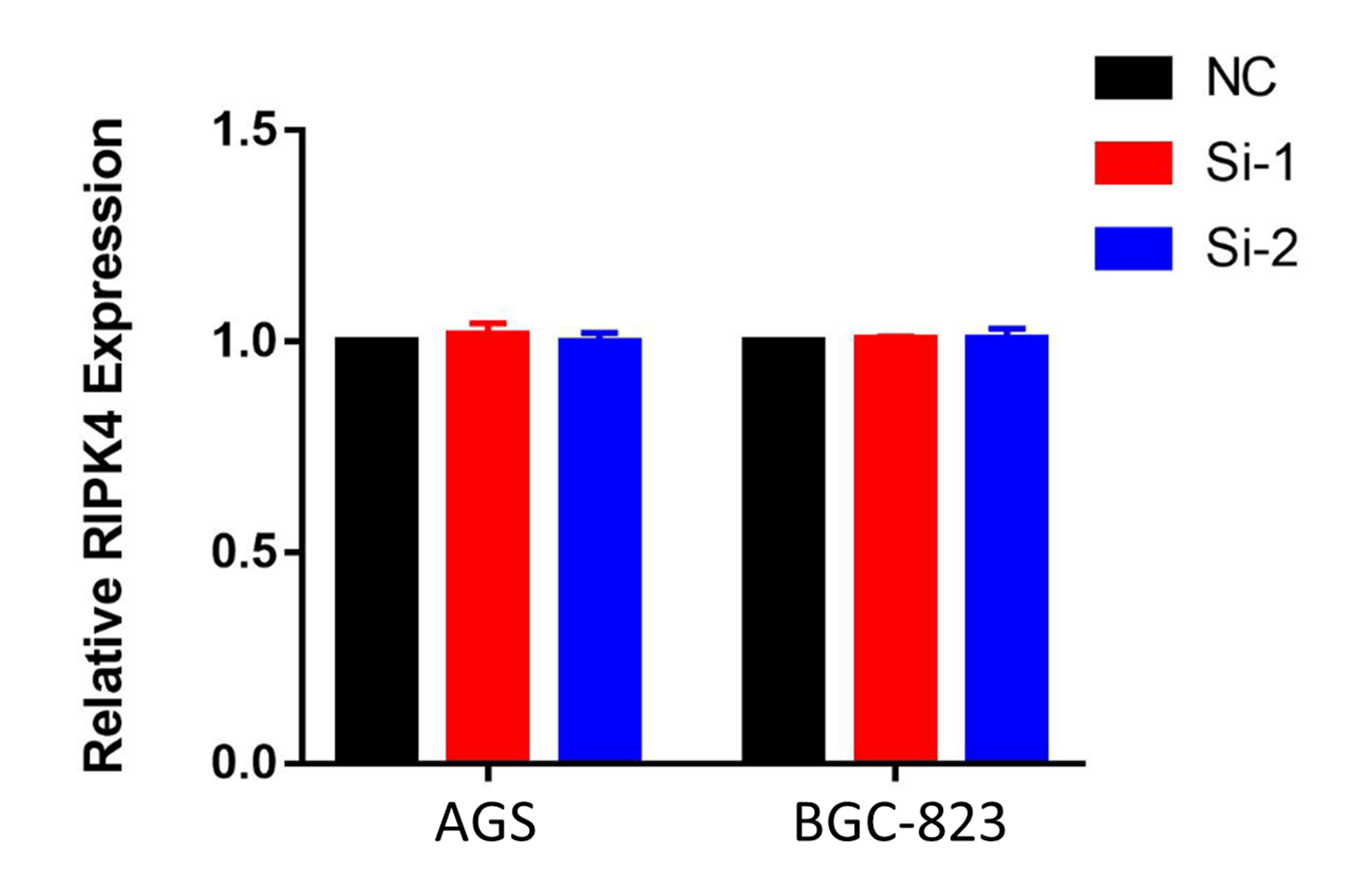

Supplement: Supplementary file 1 [file cancers-14-05237-s001.zip › Figure S5.tif]

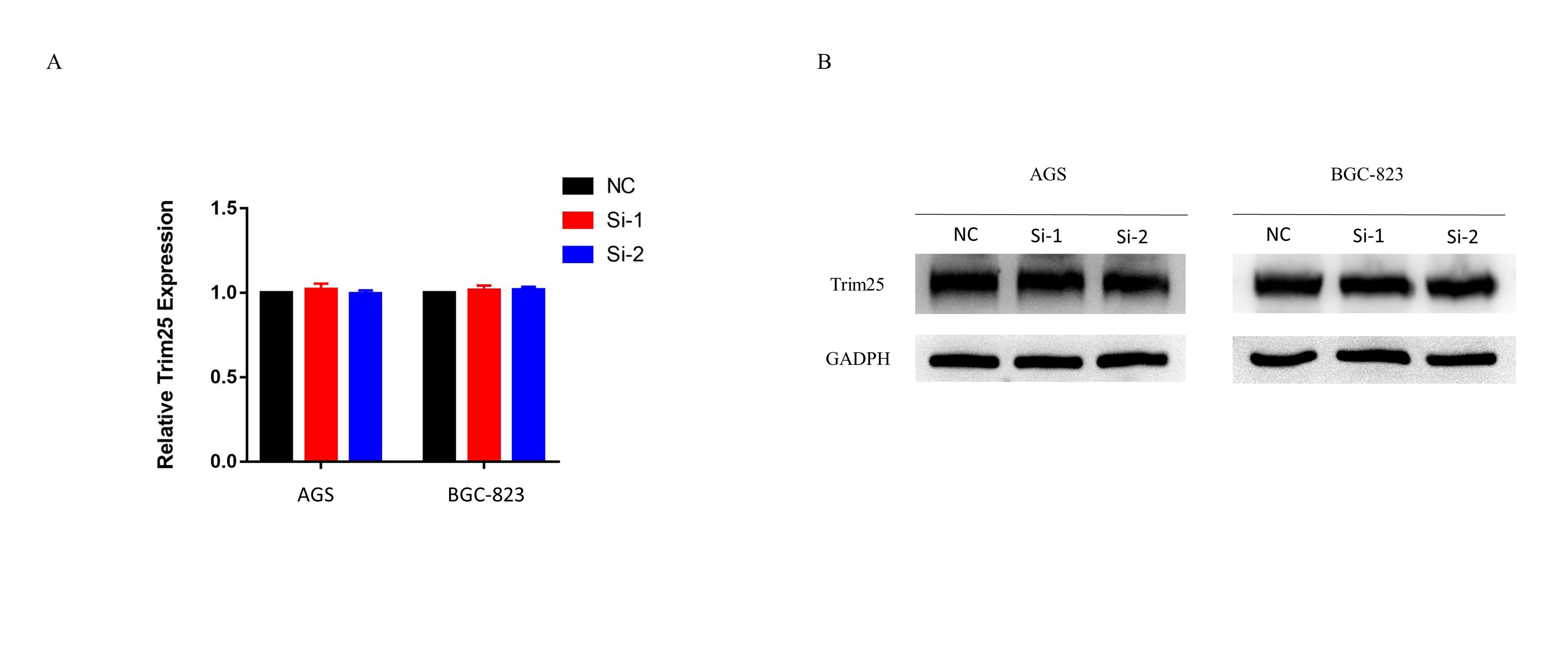

Supplement: Supplementary file 1 [file cancers-14-05237-s001.zip › Figure S6.tif]

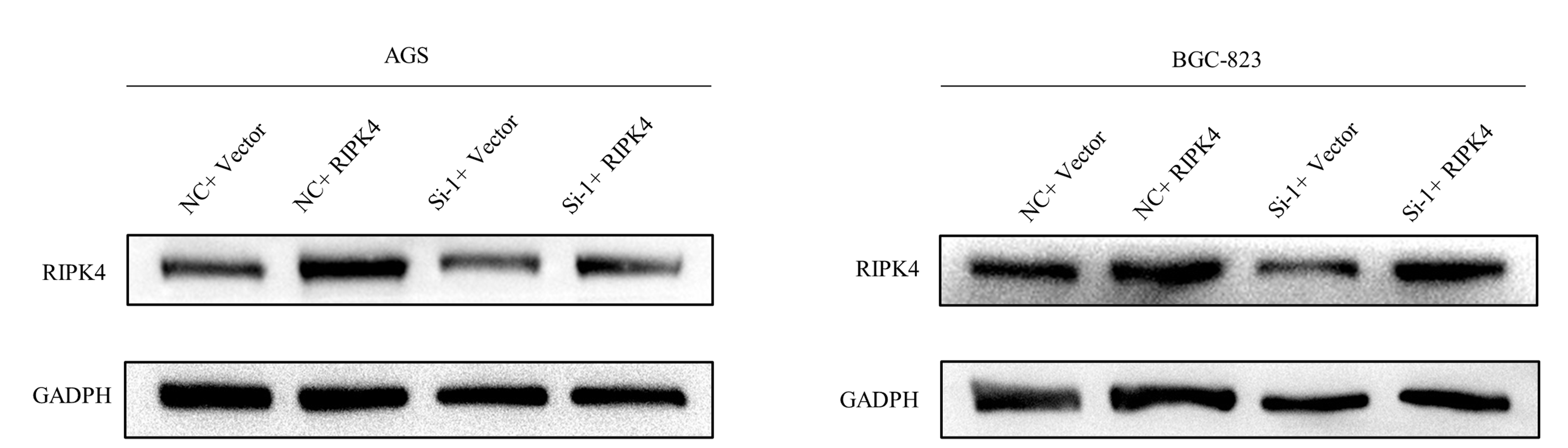

Supplement: Supplementary file 1 [file cancers-14-05237-s001.zip › Figure S7.tif]

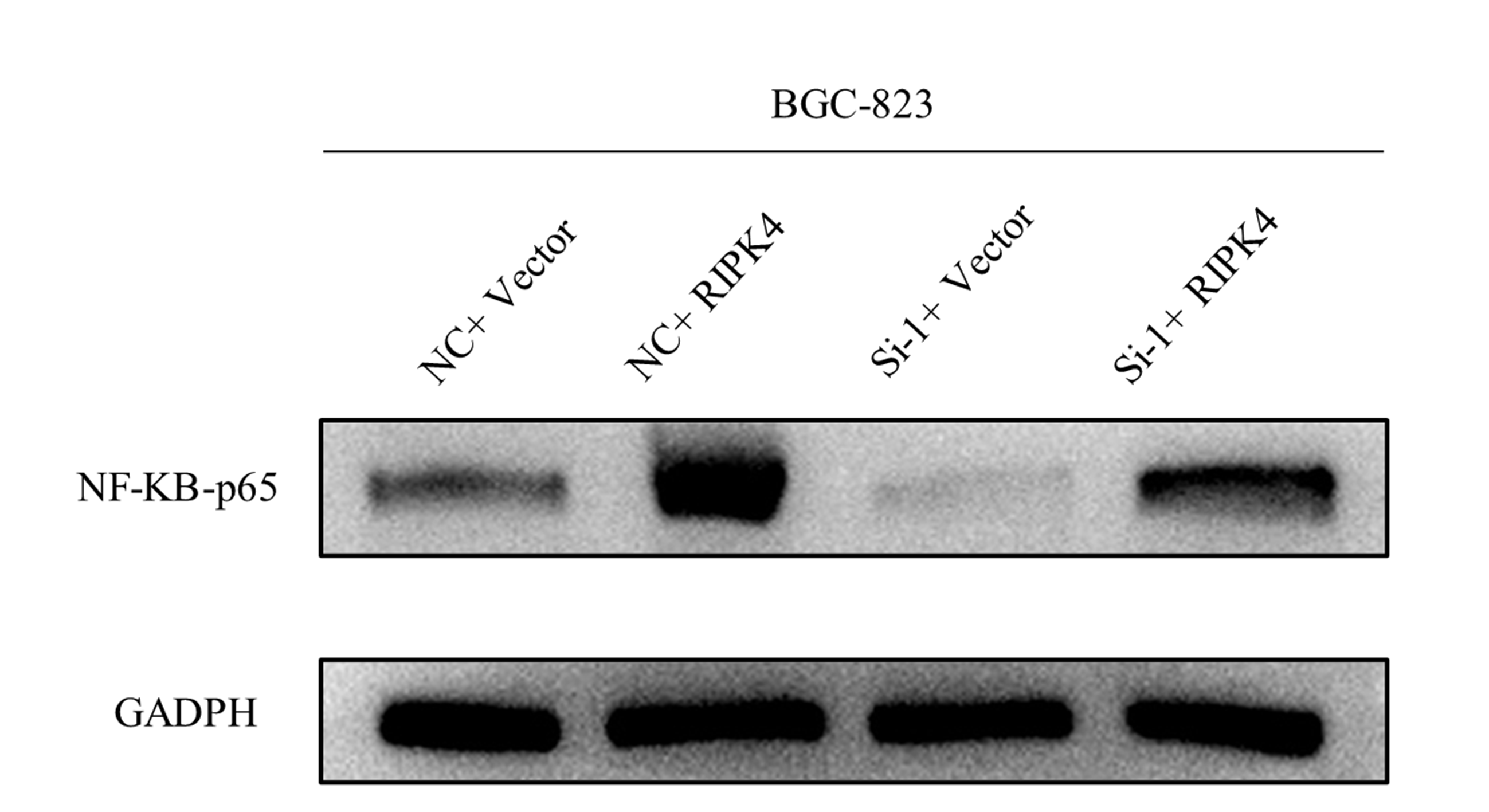

Supplement: Supplementary file 1 [file cancers-14-05237-s001.zip › Figure S8.tif]

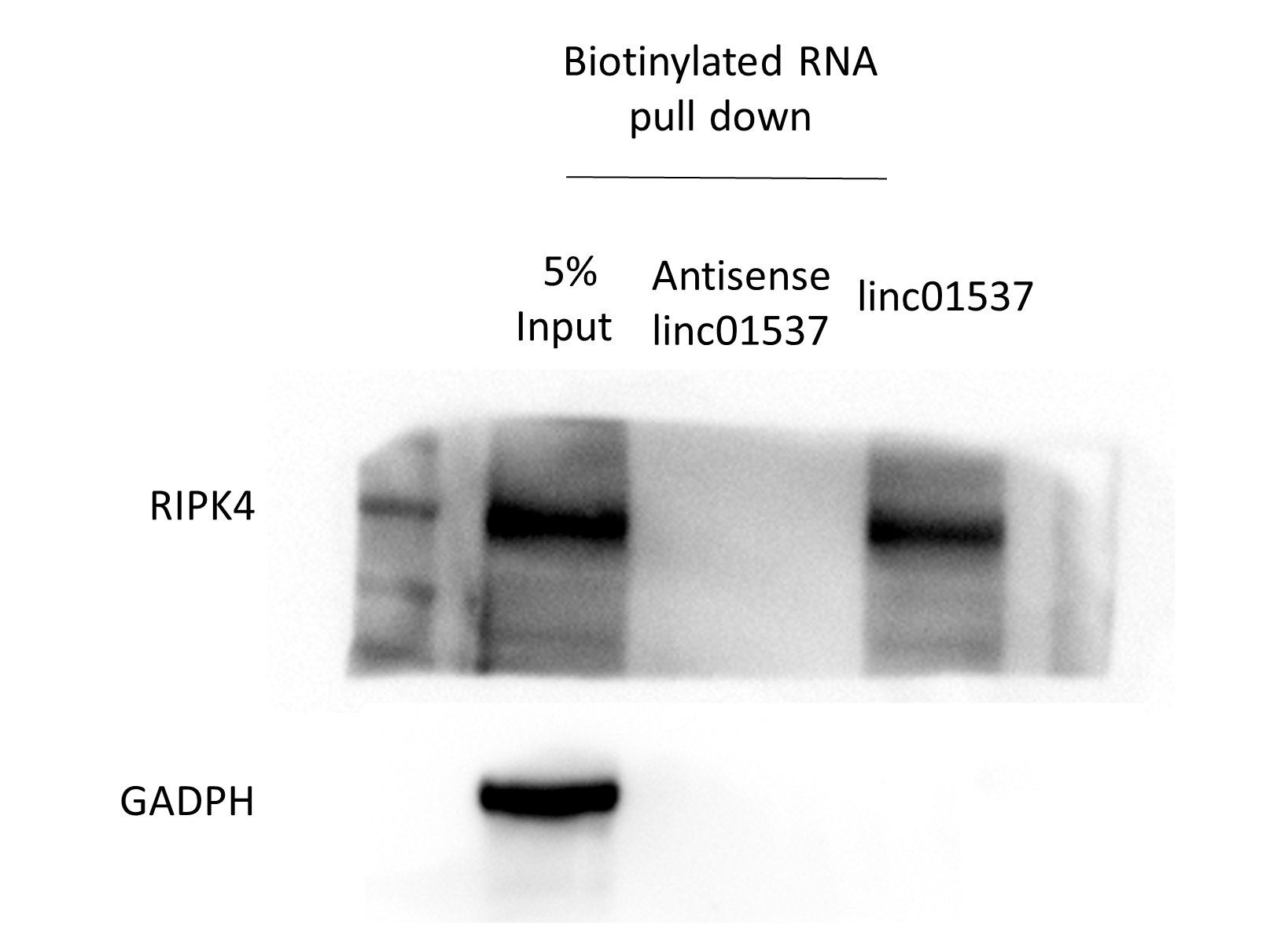

Supplement: Supplementary file 1 [file cancers-14-05237-s001.zip › Source Western-blot Images for Figure 4C.tif]

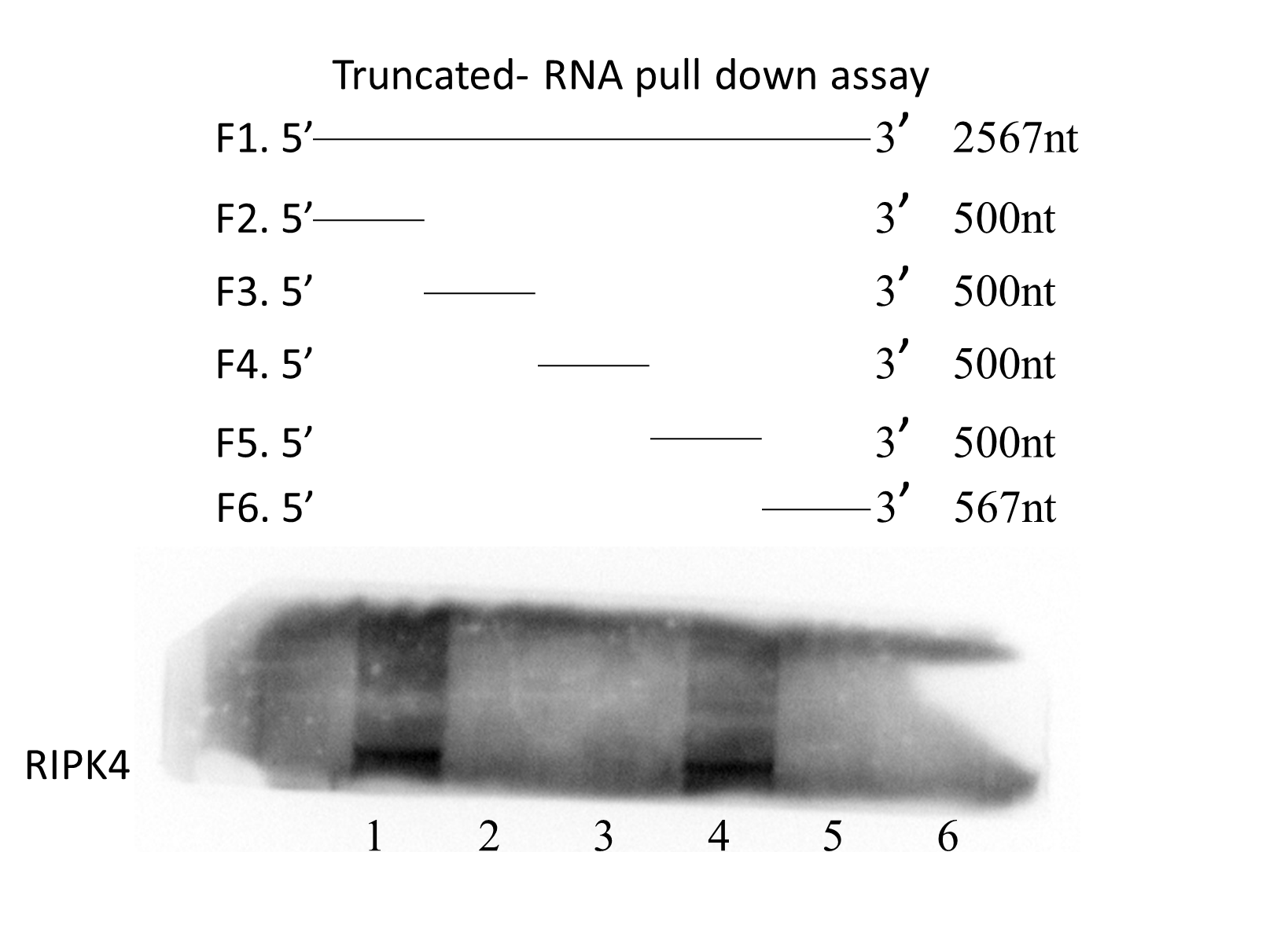

Supplement: Supplementary file 1 [file cancers-14-05237-s001.zip › Source Western-blot Images for Figure 4D.tif]

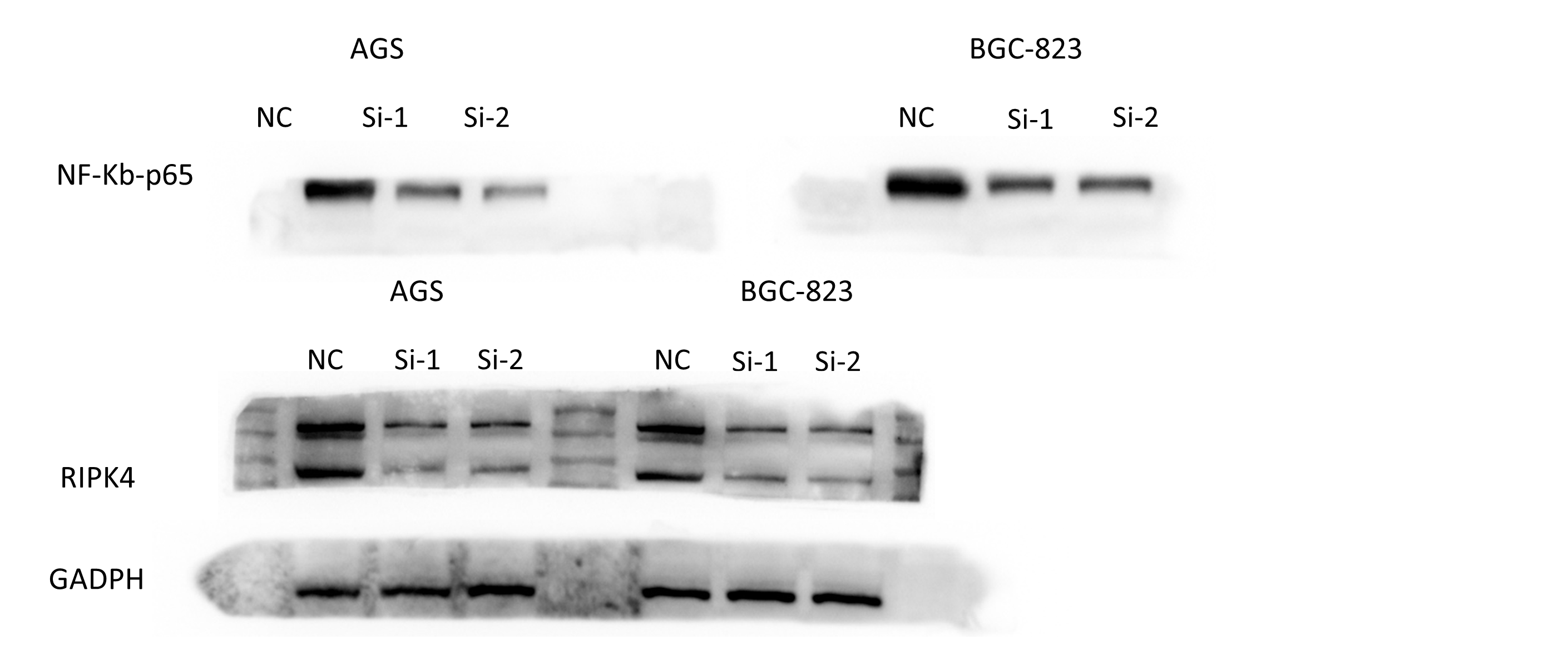

Supplement: Supplementary file 1 [file cancers-14-05237-s001.zip › Source Western-blot Images for Figure 4F.tif]

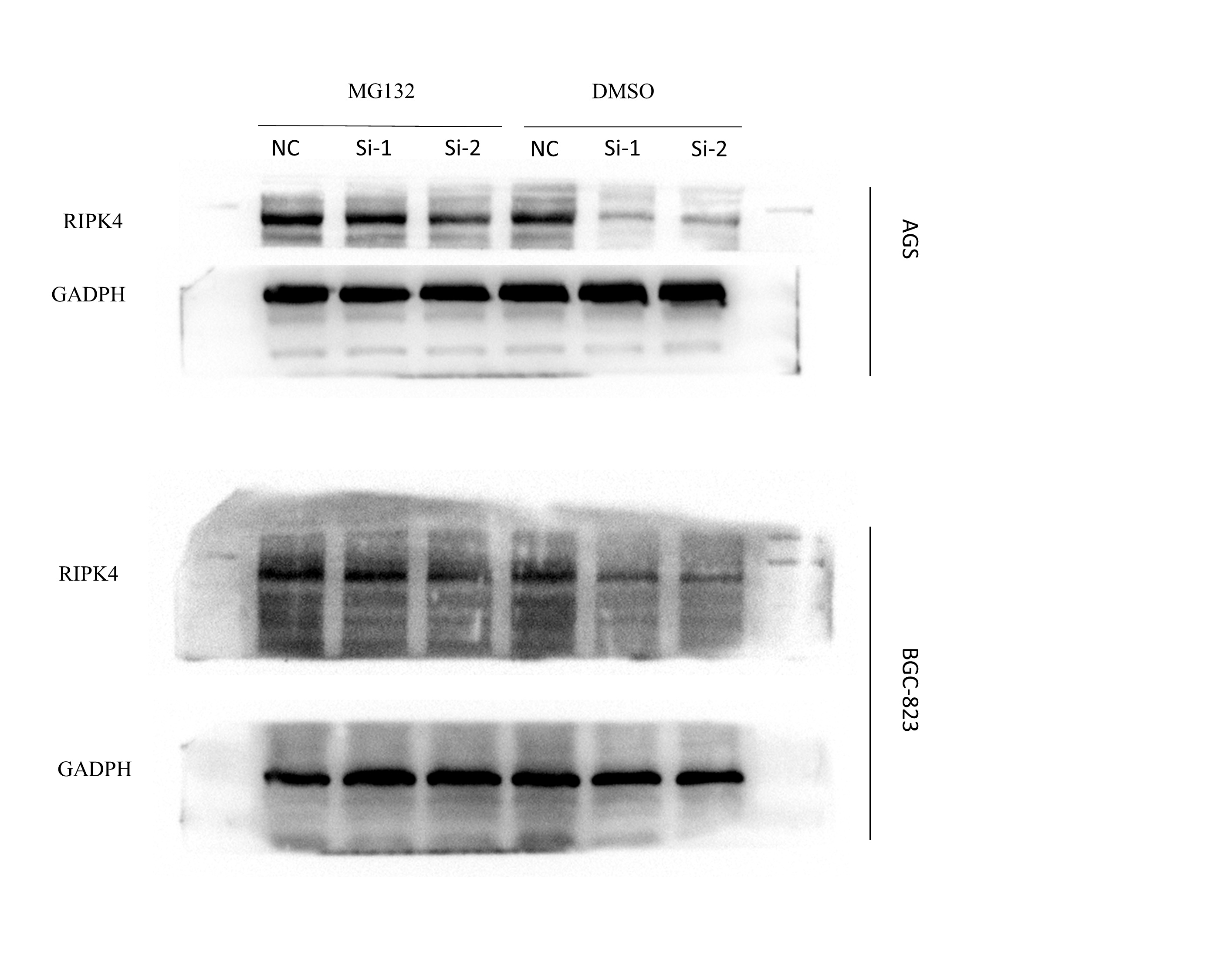

Supplement: Supplementary file 1 [file cancers-14-05237-s001.zip › Source Western-blot Images for Figure 4G.tif]

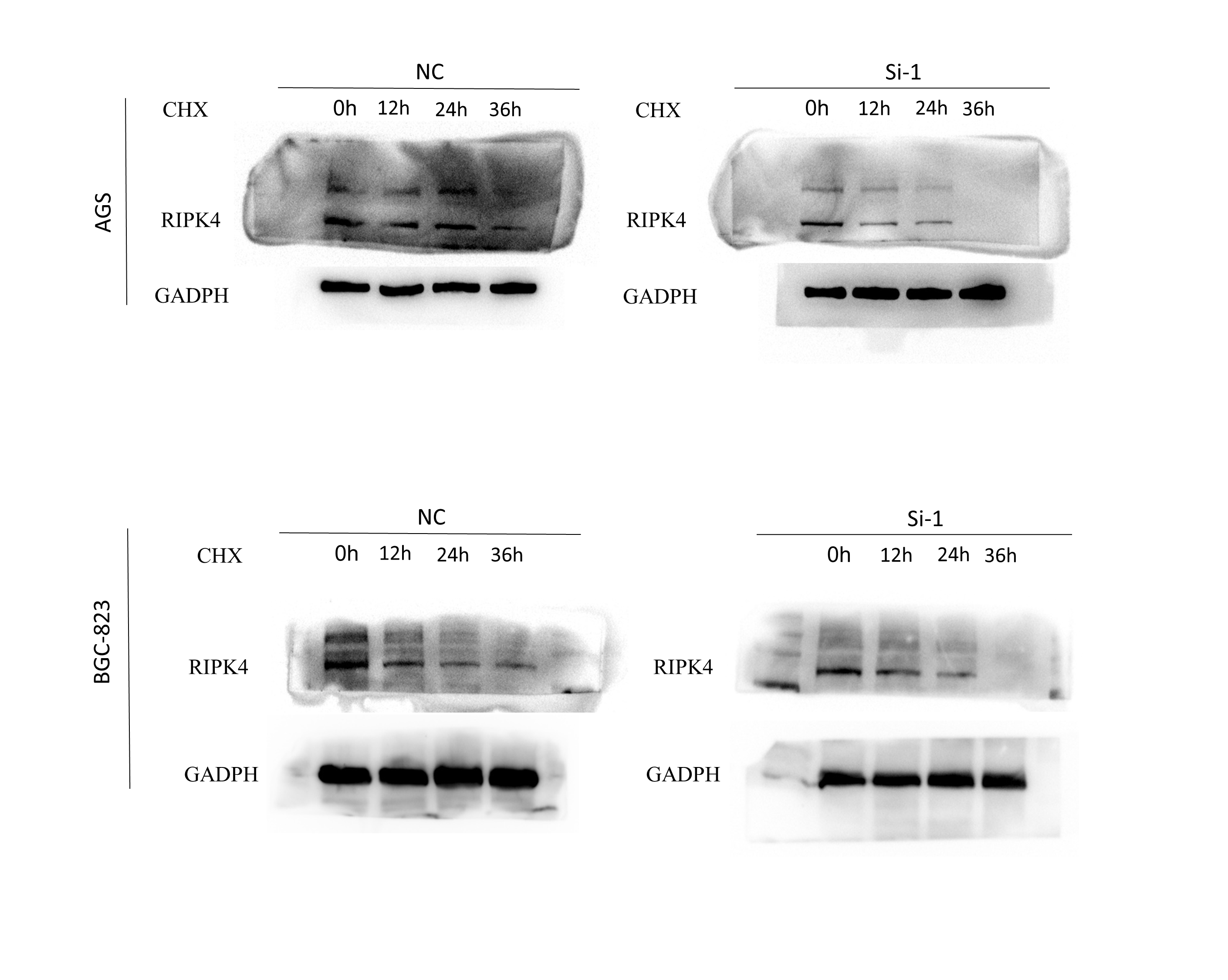

Supplement: Supplementary file 1 [file cancers-14-05237-s001.zip › Source Western-blot Images for Figure 4H.tif]

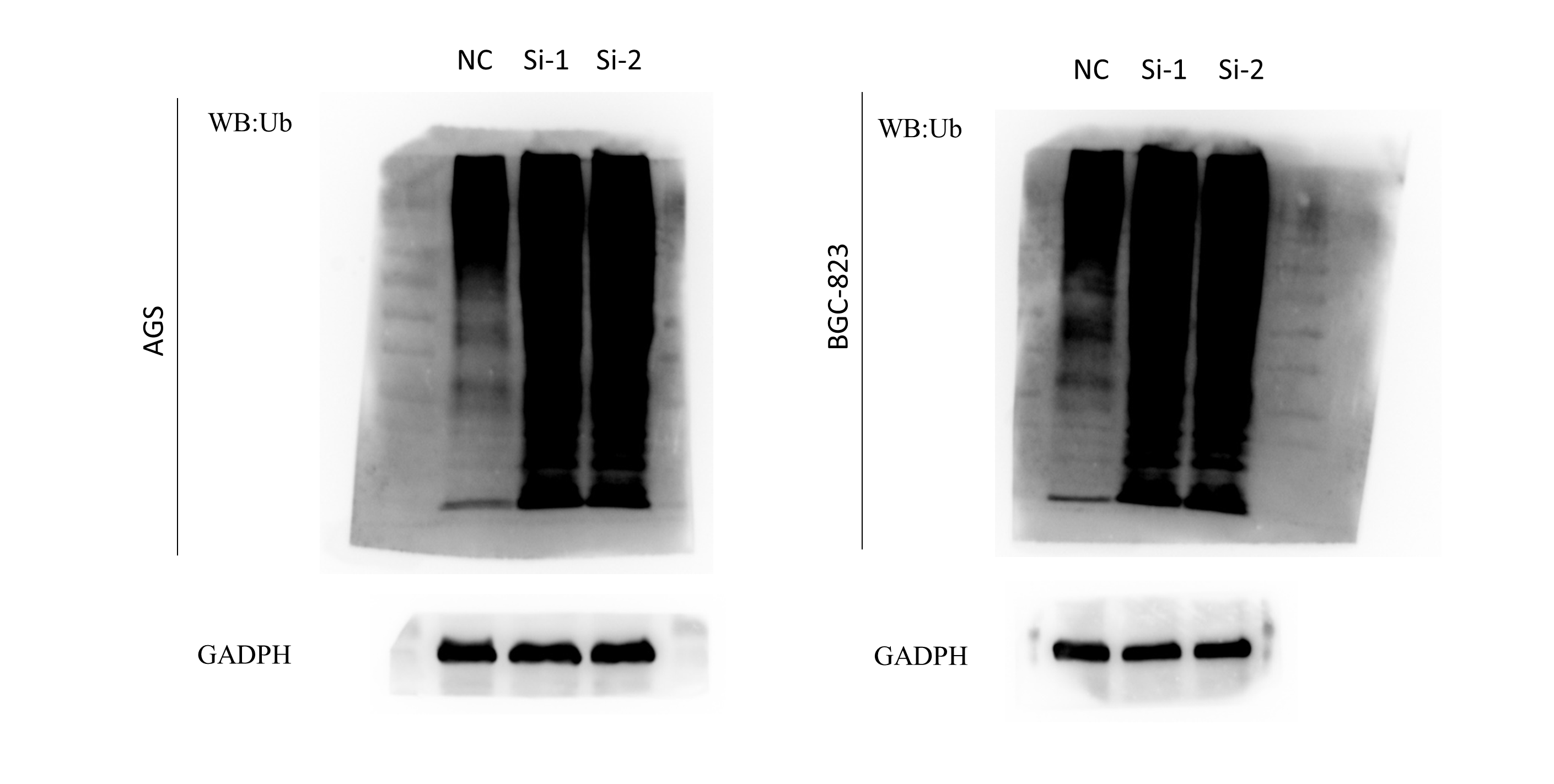

Supplement: Supplementary file 1 [file cancers-14-05237-s001.zip › Source Western-blot Images for Figure 4I.tif]

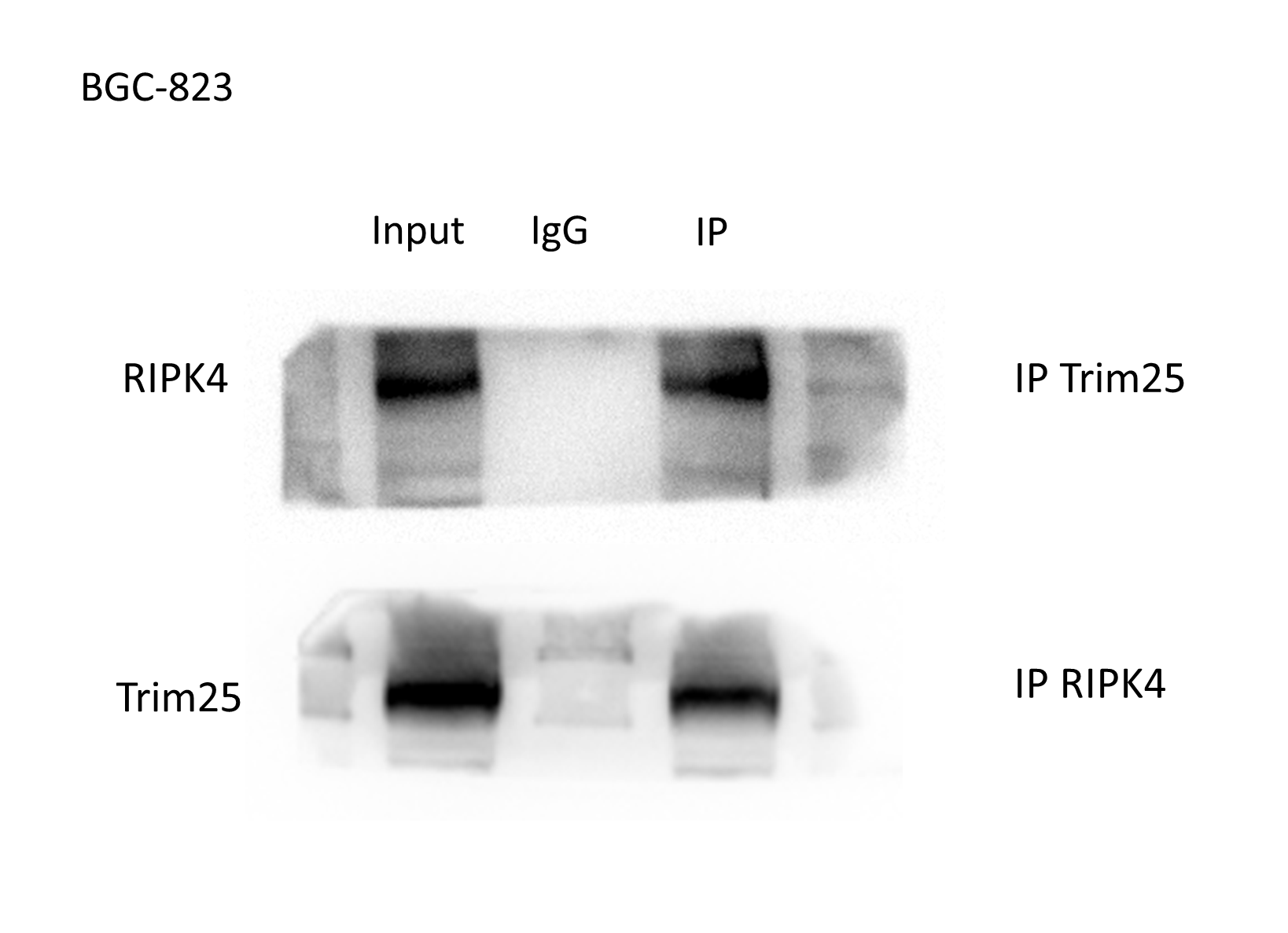

Supplement: Supplementary file 1 [file cancers-14-05237-s001.zip › Source Western-blot Images for Figure 4L.tif]

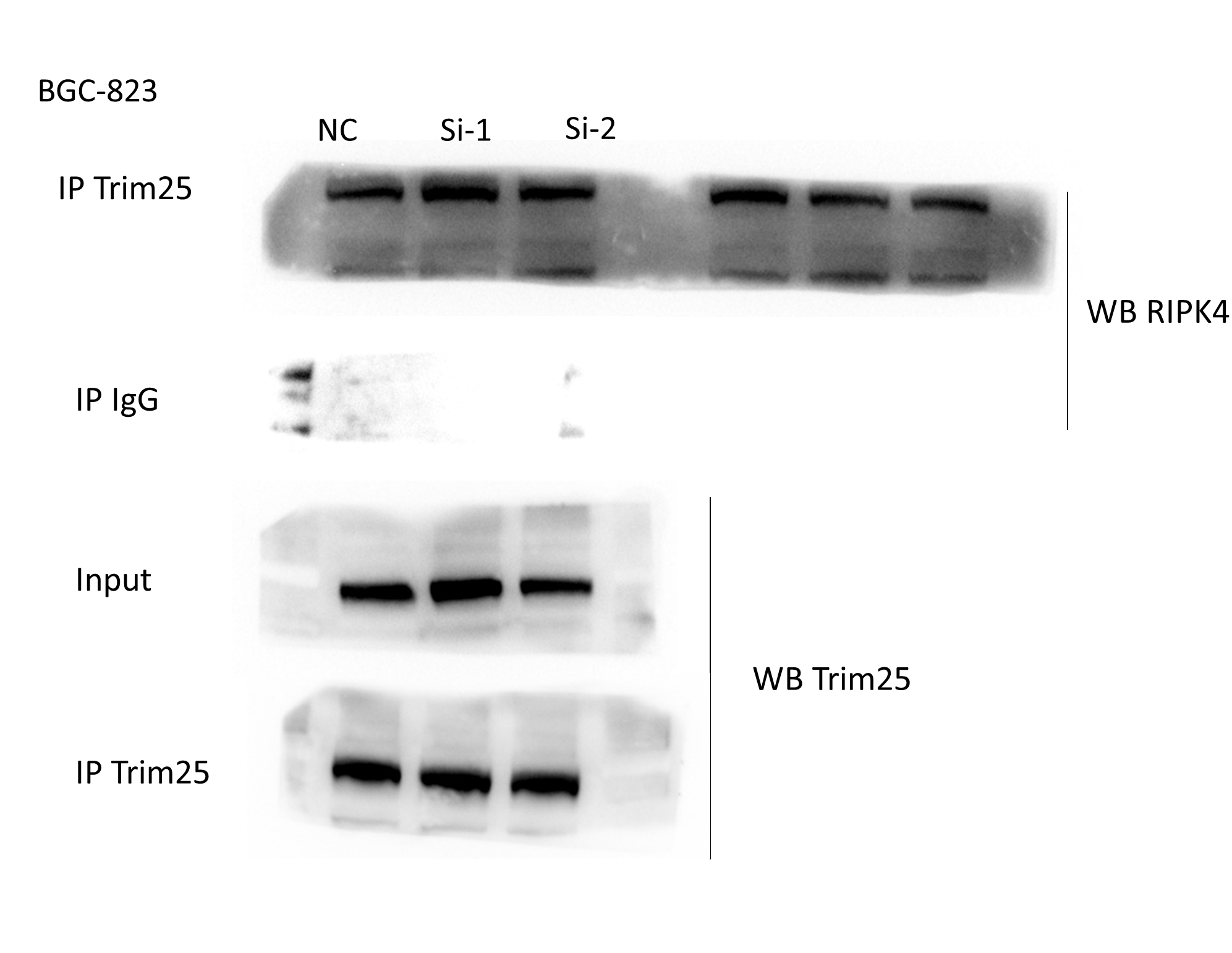

Supplement: Supplementary file 1 [file cancers-14-05237-s001.zip › Source Western-blot Images for Figure 4M.tif]

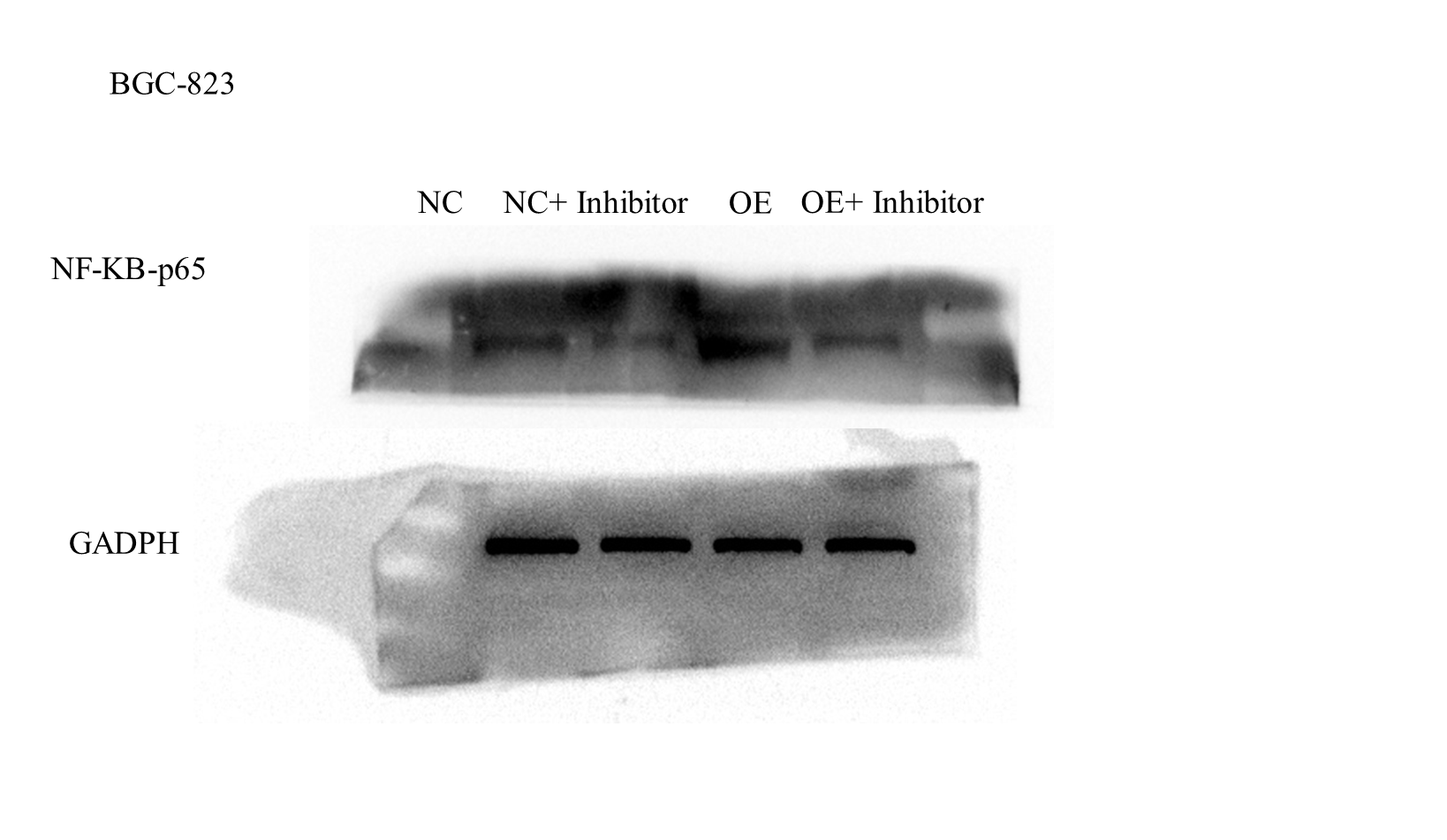

Supplement: Supplementary file 1 [file cancers-14-05237-s001.zip › Source Western-blot Images for Figure 6A.tif]

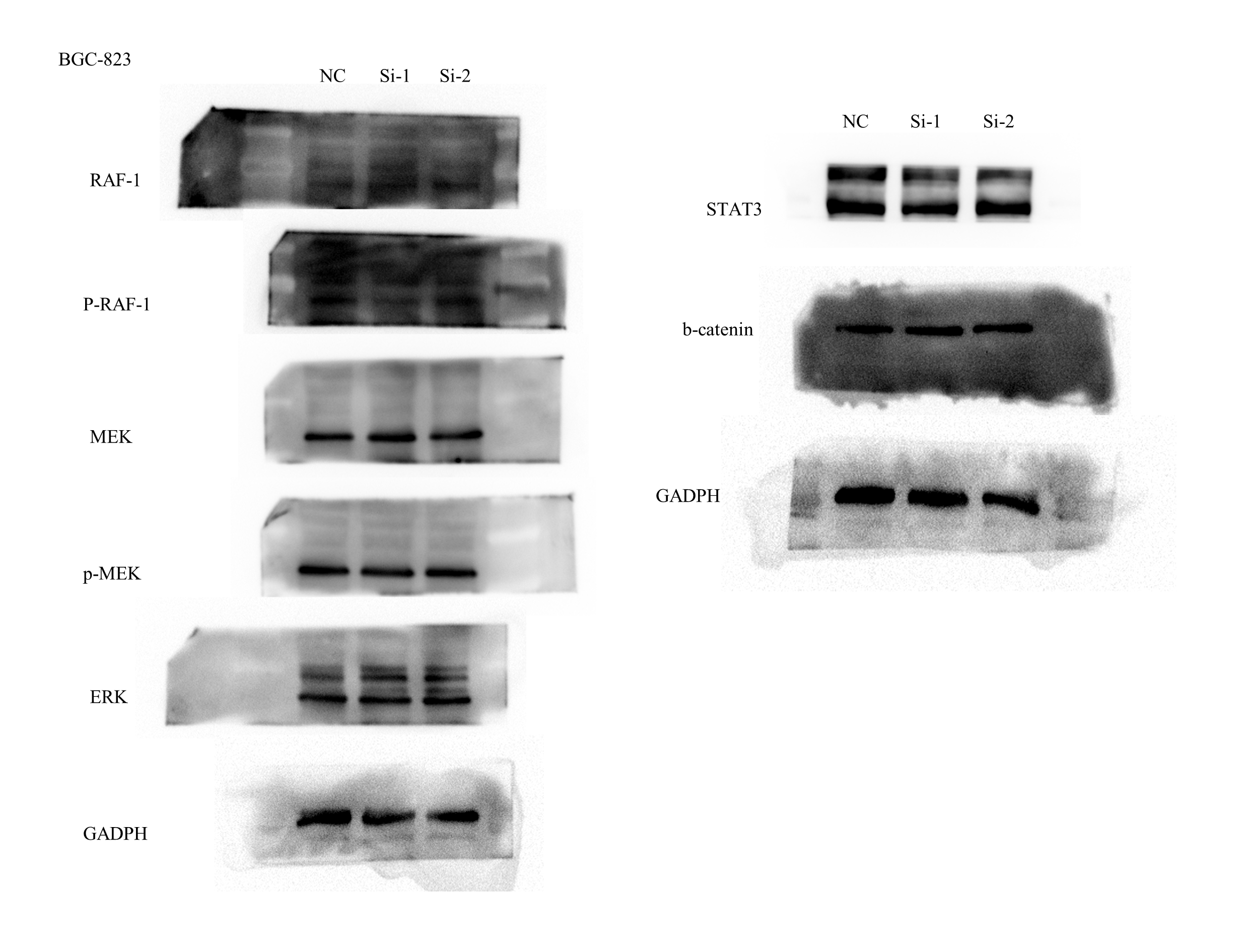

Supplement: Supplementary file 1 [file cancers-14-05237-s001.zip › Source Western-blot Images for Figure S4.tif]

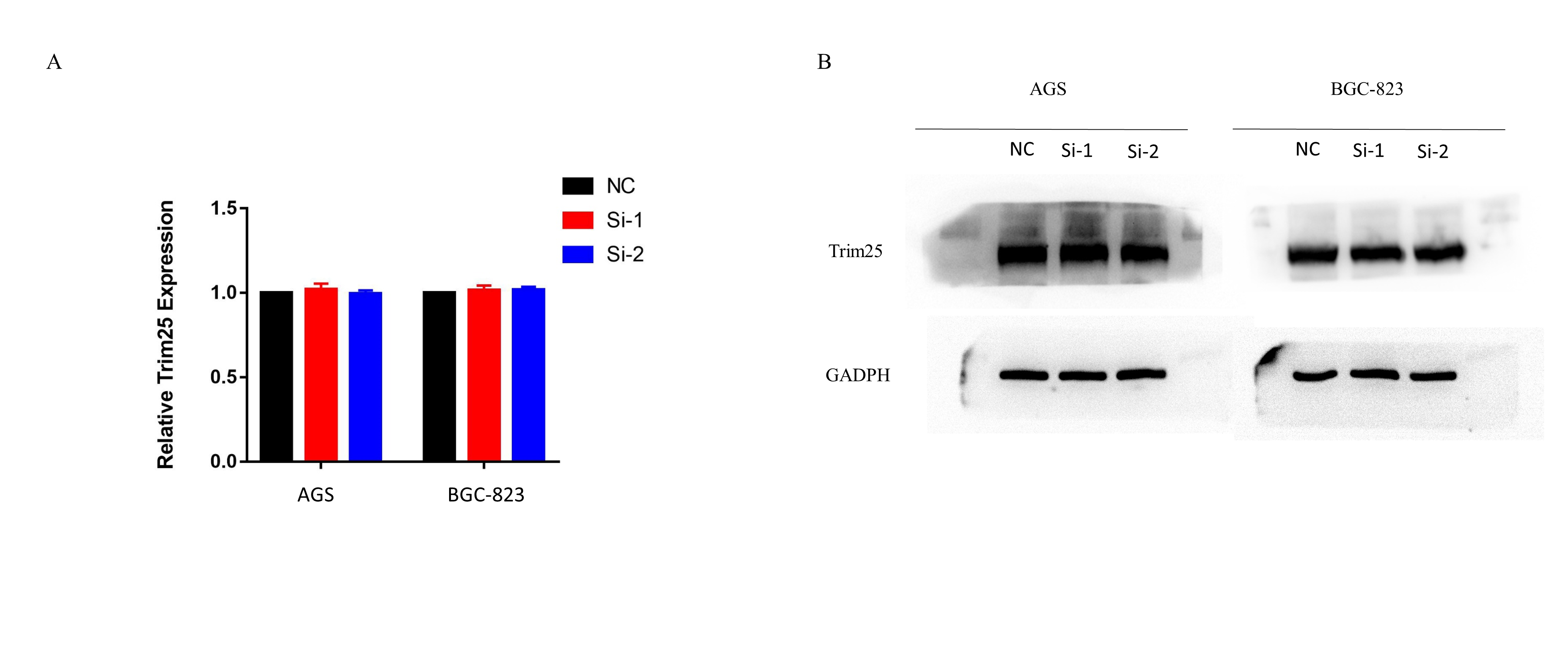

Supplement: Supplementary file 1 [file cancers-14-05237-s001.zip › Source Western-blot Images for Figure S6.tif]

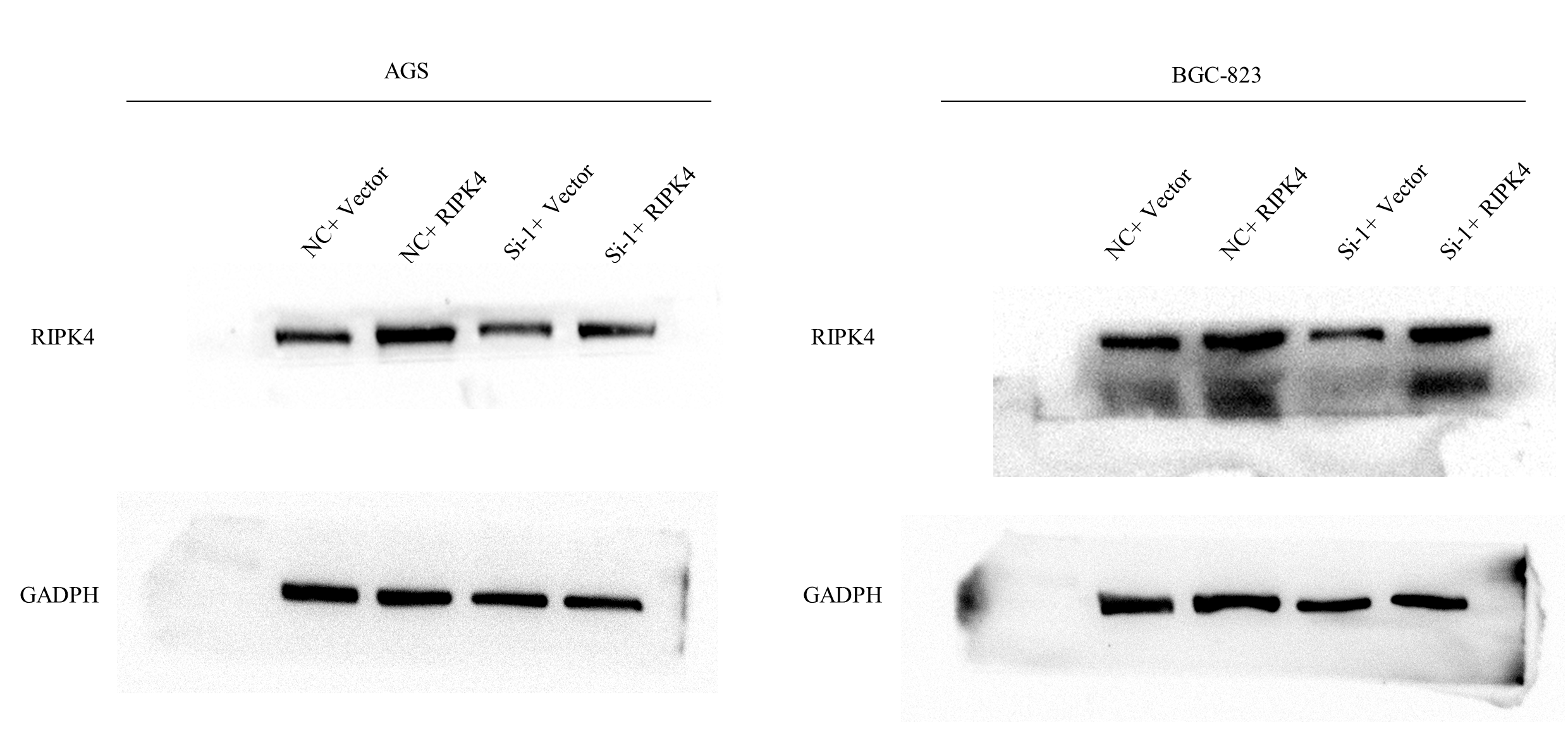

Supplement: Supplementary file 1 [file cancers-14-05237-s001.zip › Source Western-blot Images for Figure S7.tif]

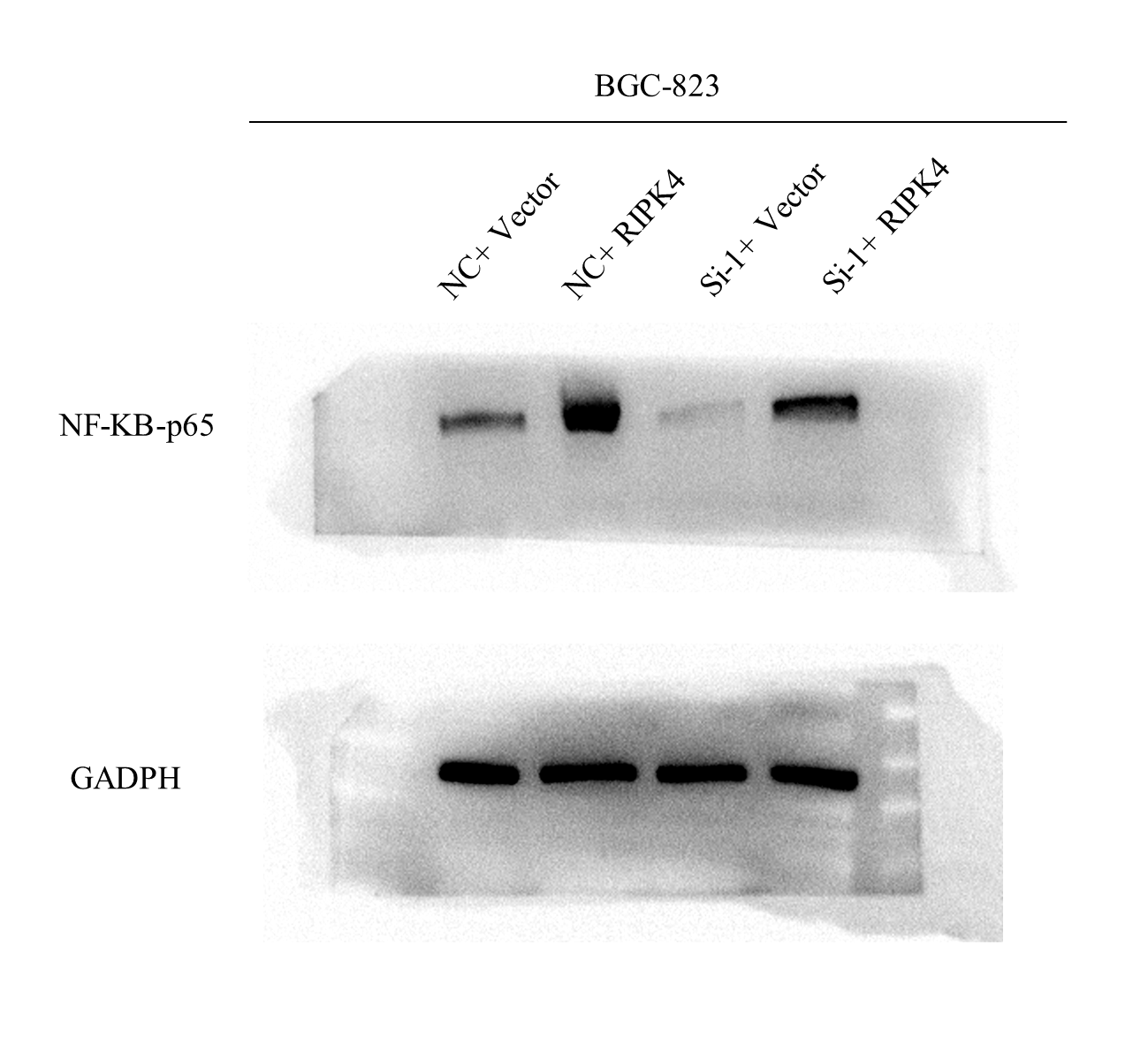

Supplement: Supplementary file 1 [file cancers-14-05237-s001.zip › Source Western-blot Images for Figure S8.tif]
